# A new species of *Trichieurina* (Diptera, Chloropidae) with a key to the world species of the genus

**DOI:** 10.3897/zookeys.433.7847

**Published:** 2014-08-13

**Authors:** Štěpán Kubík, Miroslav Barták

**Affiliations:** 1 Department of Zoology and Fisheries, Faculty of Agrobiology, Food and Natural Resources, Czech University of Life Sciences Prague, CZ-16521 Prague 6-Suchdol, Czech Republic

**Keywords:** *Trichieurina*, new species, Zambia, key, distribution

## Abstract

*Trichieurina haladai* **sp. n.** (Diptera, Chloropidae), is described from Zambia. All known *Trichieurina* species are keyed and main differential characters are illustrated.

## Introduction

The genus *Trichieurina* Duda, 1933 is a small genus of the subfamily Chloropinae (Diptera, Chloropidae) belonging to the *Platycephala* genus group sensu Andersson (1977). Until now only three species have been known in the world fauna. The first species, *Trichieurina pubescens*, was described by Meigen (1830) as *Eurina pubescens* and it is widespread in the Palaearctic region. The second species, *Trichieurina crinita*, was described by Nartshuk (1966). This species is known from Kyrgyzstan. The third species, *Trichieurina sabroskyi*, was described by Stuckenberg (1982) from Malawi. Recently we found one additional new Afrotropical species in the material of Chloropidae collected by J. Halada in Zambia.

## Material and method

The material studied is deposited in the collections of the Czech University of Life Sciences, Prague.

The genitalia were macerated in 10% KOH (24 hours, room temperature) and later stored together with specimens on plastic tag and fixed with butyl-methacrylate copolymer of methyl-methacrylate, xylene. The morphological terms used here follow Merz and Haenni (2000) and Sinclair (2000).

## Taxonomic account

### Trichieurina

Taxon classificationAnimaliaDipteraChloropidae

Duda

Trichieurina Duda, 1933: 126. Type species: *Eurina pubescens* Meigen, 1830 by original designation.

#### Redescription of the genus.

Medium sized species (4–6 mm) brown to brownish gray coloured, with strongly projecting frons, body covered with very thick brown and silvery-gray microtrichosity, and with extremely long and slender setae and setulae. Head brown, longer than high, triangular. Eyes small rounded oval, sparsely covered with long setulae. Gena broad, equally broad behind as height of compound eye, brown with gray microtrichosity and long dark setulae. Postgena gray microtrichose, lower margin brownish black or yellow without microtrichosity. Vibrissa indistinct. Frons projecting before eye margin at a distance corresponding to the length of eye (*Trichieurina pubescens* and African species) or half length of eye (*Trichieurina crinita*), with triangular fore margin and long frontal setae. Hind margin of ocellar triangle as broad as half width of frons, with straight lateral margins, reaching front of frons (Fig. 7) or narrowing to middle and reaching front of frons as the narrow point, covered by thick brown to gray microtrichosity and with longitudinal median groove (Figs 5, 6). Interfrontal setae arranged in one or two rows along margin of ocellar triangle, long, irregularly incurved. Ocellar and vertical setae long and slender. Internal and external vertical setae indistinct, about six orbital setae. Clypeus large, brown to black and microtrichose, proboscis short, palpus short and dark or yellow. Antenna brown or yellow, pedicel large, about as long as broad, 1^st^ flagellomere longer than broad. Arista almost naked, white, dark on basal segment.

Thorax dark brown, entirely covered with gray or brown microtrichosity. Postpronotal lobe with numerous dark long setulae (no distinct setae). Scutum convex, covered with gray microtrichosity and with numerous long dark setulae. Notopleuron with a group of long dark setulae and indistinct setae. Scutellum rounded, convex, microtrichose, brown in middle, gray at sides and with long dark setulae. Apical and lateral scutellar setae long and dark. Pleuron completely microtrichose. Anepisternum and anepimeron without setulae, katepisternum covered with long dark or pale setulae.

Legs simple, gray microtrichose. Tibial organ absent.

Wings brown with thick dark brown veins. Halter dark brown or yellow.

Abdomen oval, dorsally covered with brown microtrichosity, gray microtrichose at sides and below and with numerous long dark setulae.

Male genitalia rather similar to those of *Platycephala* and *Eurina*, surstylus short and broad.

### Trichieurina haladai sp. n.

Taxon classificationAnimaliaDipteraChloropidae

http://zoobank.org/FA6793CE-8429-4072-8A20-288CCCA73F7C

**Figure 1–2.**
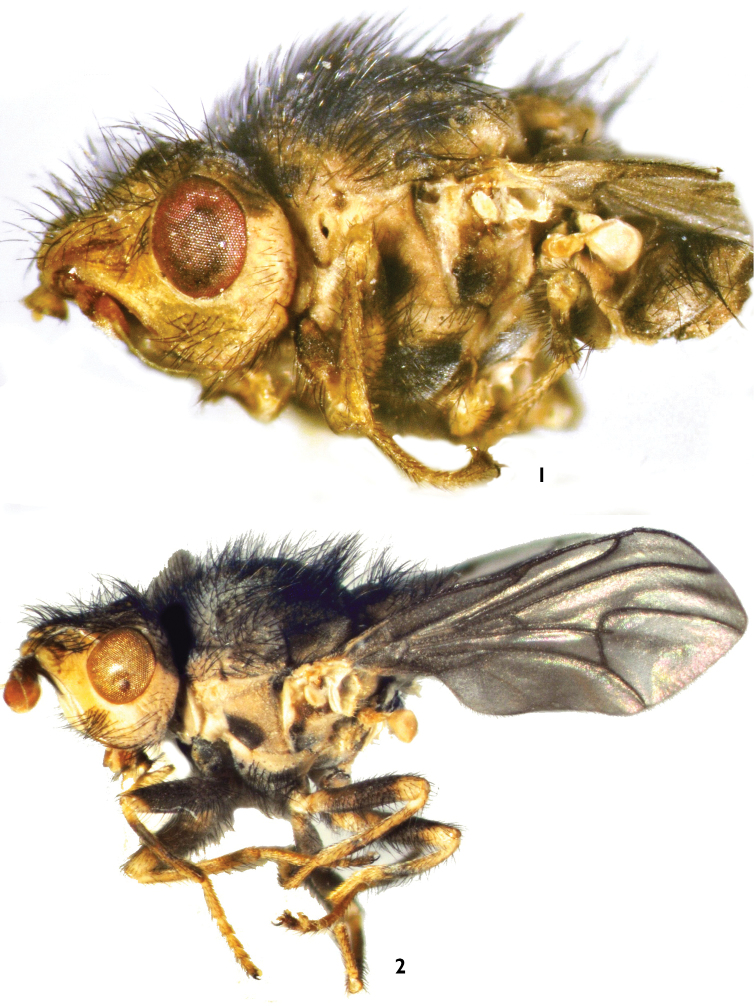
**1** *Trichieurina haladai* sp. n. (holotype): lateral view **2** *Trichieurina sabroskyi*: lateral view.

**Figure 3–8.**
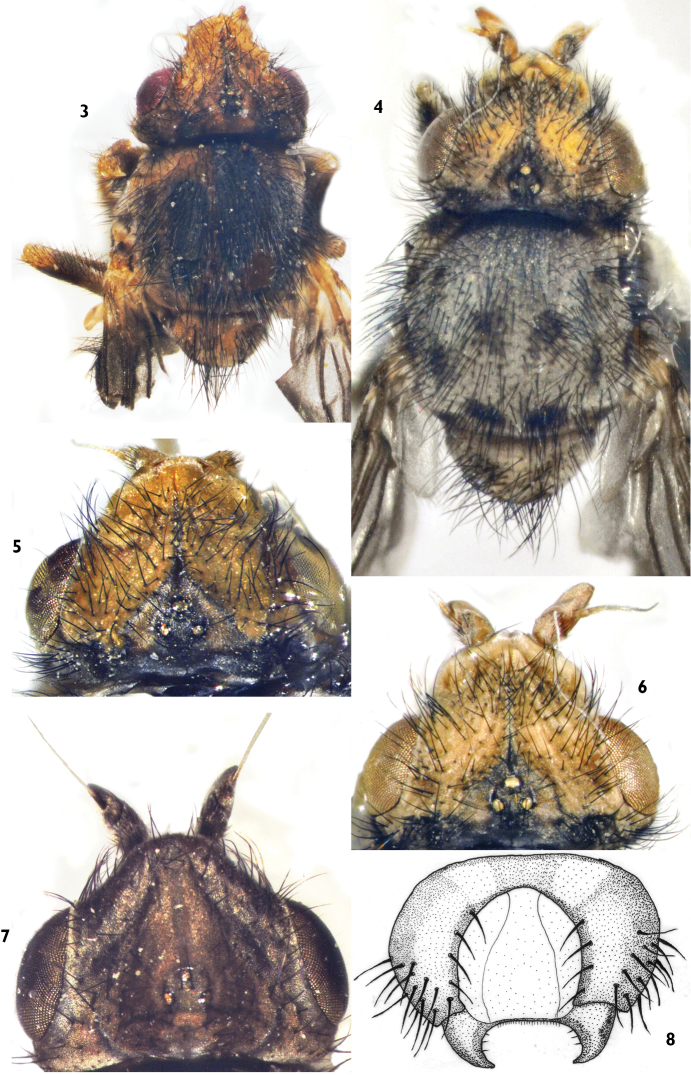
**3** *Trichieurina haladai* sp. n. (holotype): dorsal view **4** *Trichieurina sabroskyi*: dorsal view **5** *Trichieurina haladai* sp. n. (holotype): Head with ocellar triangle **6** *Trichieurina sabroskyi*: Head with ocellar triangle **7** *Trichieurina pubescens*: Head with ocellar triangle **8** *Trichieurina haladai* sp. n. (holotype): epandrium.

Figs 1 , 3 , 5 , 8 , 10 , 12

#### Material examined.

Holotype: male, Zambia NW, Solwezi env. 12°11'40"S, 26°23'56"E, 1.–3.xii.2002, J. Halada leg. Paratype: female, Zambia C, 120 km N from Lusaka, 14°19'40"S, 28°16'56"E, 12.–14.xii.2002, J. Halada leg. Holotype and paratype are deposited in the collection of the Czech University of Life Sciences Prague.

#### Diagnosis.

Similar to *Trichieurina sabroskyi* in the shape and colour of ocellar triangle, but the scutum is yellowish brown with three broad black stripes.

#### Description.

(Holotype) Body length 6 mm. Head yellowish brown, higher than long. Eye small, rounded oval and vertically elongated. Gena broad, as broad behind as the width of eye, yellow, with yellow microtrichosity and long dark setulae. Postgena with yellow lower edge without microtrichosity. Frons projecting before eye margin at a distance corresponding to the length of eye, with triangular fore margin and long black frontal setae. Ocellar triangle posteriorly as broad as half width of frons, narrowing to middle and reaching front of frons as a narrow point, brown, with gray microtrichosity and with dark brown longitudinal median groove (Fig. 5). Ocellar tubercle black. Interfrontal setae in two rows on margin of ocellar triangle, long, irregularly incurved. Ocellar setae long and slender, similar to other relatively long setulae on head. External and internal vertical setae indistinct, numerous orbital setae. Clypeus large, brown to black, slightly gray microtrichose. Proboscis short, palpus short, but clubbed, yellow, with numerous long black setulae. Antenna yellow, pedicel large, about as long as broad, 1^st^ flagellomere longer than broad. Arista almost naked, white, with yellow basal segment.

Thorax yellowish–brown, completely covered with brownish yellow or gray microtrichosity. Postpronotal lobe brown with dark rounded spot and numerous long black setulae, postpronotal setae indistinct. Scutum convex, brown, with three broad blackish brown and gray microtrichose stripes and with numerous very long black setae. Notopleuron with numerous long dark setae. Scutellum rounded, yellow, with yellow microtrichosity and long dark setae. Apical and lateral scutellar setae long and black. Pleuron completely microtrichose. Both anepisternum and anepimeron yellow each with dark rounded spot and without setulae, katepisternum yellow with large black and silvery microtrichose spot and covered with numerous dark setulae. Legs simple, yellowish brown with gray microtrichosity.

Wings brown with thick dark brown veins. Halter yellow.

Abdomen oval, yellowish brown, microtrichose and with numerous long setulae.

**Figure 9–13.**
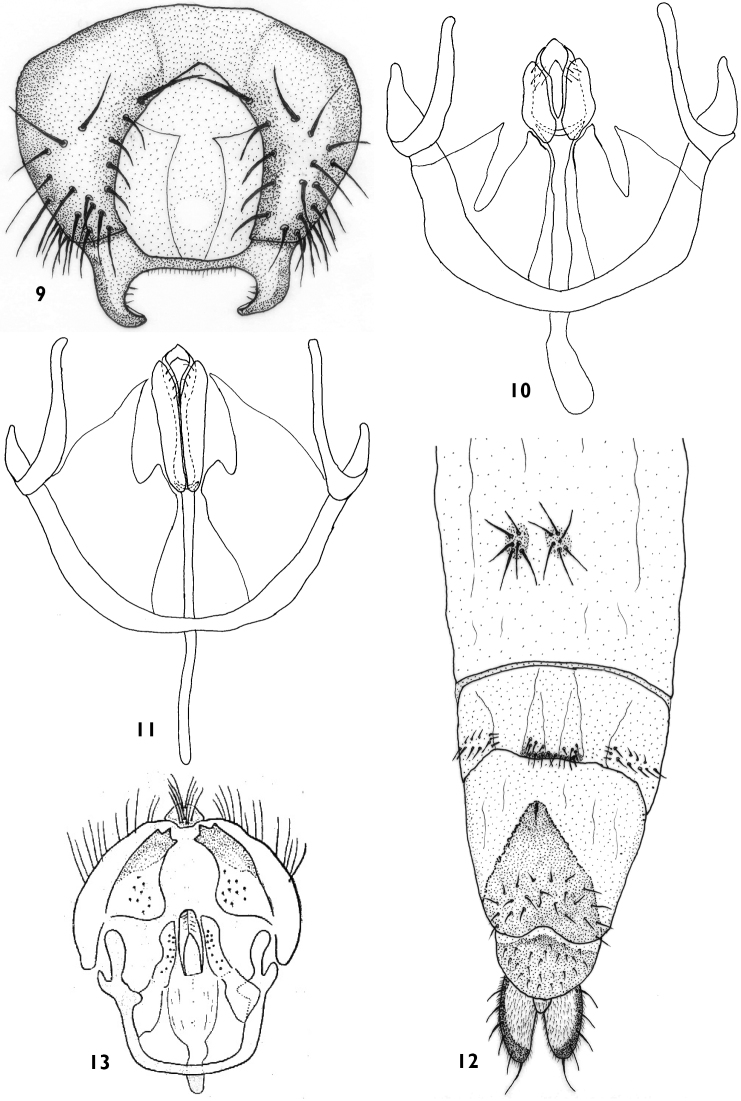
**9** *Trichieurina sabroskyi*: epandrium **10** *Trichieurina haladai* sp. n. (holotype): hypandrium and phallic complex **11** *Trichieurina sabroskyi*: hypandrium and phallic complex **12** *Trichieurina haladai* sp. n. (paratype): female terminalia **13** *Trichieurina crinita*: male genitalia (after Nartshuk 1966).

Male genitalia (Figs 8 and 10) similar to *Trichieurina sabroskyi* (Figs 9 and 11), epandrium of *Trichieurina haladai* (Fig. 8) broader and more rounded, surstylus broader and more pointed, thorn–hooked. Hypandrium (Fig. 10) open, postgonite broad and short.

Female with long cerci (Fig. 12).

#### Etymology.

The species is named in honor of J. Halada, a collector of the types.

### Key to world species of *Trichieurina* Duda, 1933

**Table:** 

1	Eye longer than high. Ocellar triangle evenly narrowing towards tip (Fig. 7). Ground colour of body gray, scutum with two narrow brownish-black median stripes	*Trichieurina pubescens*
–	Eye higher than long or rounded. Ocellar triangle abruptly narrowing towards middle, forming narrow line in anterior half. Ground colour yellow or yellowish brown, scutum either with irregular brown markings or with three longitudinal blackish–brown stripes	2
2	Frons projecting before eye margin at a distance corresponding to half length of eye, phallapodeme broad, surstylus massive with three tooth-like projections on top (Fig. 13)	*Trichieurina crinita*
–	Frons projecting before eye margin at a distance corresponding to length of eye, phallapodeme thin, surstylus short and hooked	3
3	Lateral margin of ocellar triangle straight in basal third (Fig. 5), scutum yellowish–brown with three broad black stripes (Fig. 3), male genitalia as in Figs 8 and 10, surstylus shorter, thorn-hooked, postgonites short and wider	*Trichieurina haladai* sp. n.
–	Lateral margin of ocellar triangle convex in basal third (Fig. 6), scutum brown with irregular dark brown marks (Fig. 4), male genitalia as in Figs 9 and 11, surstylus longer, more U–hooked, postgonites longer and thin	*Trichieurina sabroskyi*

## Distribution

*Trichieurina haladai* sp. n. (Fig. 1): Zambia-NW, Solwezi env. 12°11'40"S, 26°23'56"E, Zambia C, 120 km N from Lusaka, 14°19'40"S, 28°16'56"E.

*Trichieurina crinita* Nartshuk, 1966: Kyrgyzstan: Kirghiz Mountain Range, pass Chaj-Sandyk, Taldy–Bulak river (Nartshuk 1966).

*Trichieurina pubescens* (Meigen, 1830): Austria, Bulgaria, Czech Republic, Finland, French mainland, Germany, Hungary, Italian mainland, Lithuania, Russia Northwest, Russia South, Ukraine, East Palaearctic region. (Nartshuk 2007).

*Trichieurina sabroskyi* Stuckenberg, 1982 (Fig. 2): Malawi (Stuckenberg 1982). Zambia, Kasempa env., 13°27'37"S, 25°50'21"E, 16.–18.xi.2006, Kubik leg (1 male), Zambia, Mumbwa env. 20 km S to Lusaka rd., 15°35'14"S, 28°16'14"E, 21.–22.xi.2006, Kubik leg. (1 male). First records from Zambia.

## Supplementary Material

XML Treatment for Trichieurina

XML Treatment for Trichieurina haladai
